# A New Methodology to Associate SNPs with Human Diseases According to Their Pathway Related Context

**DOI:** 10.1371/journal.pone.0026277

**Published:** 2011-10-25

**Authors:** Burcu Bakir-Gungor, Osman Ugur Sezerman

**Affiliations:** 1 Biological Sciences and Bioengineering, Faculty of Engineering, Sabancı University, İstanbul, Turkey; 2 Department of Genetics Bioinformatics, Department of Computer Engineering, Faculty of Engineering, Bahçeşehir University, İstanbul, Turkey; Centro de Investigación Príncipe Felipe, Spain

## Abstract

Genome-wide association studies (GWAS) with hundreds of żthousands of single nucleotide polymorphisms (SNPs) are popular strategies to reveal the genetic basis of human complex diseases. Despite many successes of GWAS, it is well recognized that new analytical approaches have to be integrated to achieve their full potential. Starting with a list of SNPs, found to be associated with disease in GWAS, here we propose a novel methodology to devise functionally important KEGG pathways through the identification of genes within these pathways, where these genes are obtained from SNP analysis. Our methodology is based on functionalization of important SNPs to identify effected genes and disease related pathways. We have tested our methodology on WTCCC Rheumatoid Arthritis (RA) dataset and identified: i) previously known RA related KEGG pathways (e.g., Toll-like receptor signaling, Jak-STAT signaling, Antigen processing, Leukocyte transendothelial migration and MAPK signaling pathways); ii) additional KEGG pathways (e.g., Pathways in cancer, Neurotrophin signaling, Chemokine signaling pathways) as associated with RA. Furthermore, these newly found pathways included genes which are targets of RA-specific drugs. Even though GWAS analysis identifies 14 out of 83 of those drug target genes; newly found functionally important KEGG pathways led to the discovery of 25 out of 83 genes, known to be used as drug targets for the treatment of RA. Among the previously known pathways, we identified additional genes associated with RA (e.g. Antigen processing and presentation, Tight junction). Importantly, within these pathways, the associations between some of these additionally found genes, such as HLA-C, HLA-G, PRKCQ, PRKCZ, TAP1, TAP2 and RA were verified by either OMIM database or by literature retrieved from the NCBI PubMed module. With the whole-genome sequencing on the horizon, we show that the full potential of GWAS can be achieved by integrating pathway and network-oriented analysis and prior knowledge from functional properties of a SNP.

## Introduction

Genome-Wide Association Studies (GWAS) – in which hundreds of thousands of single nucleotide polymorphisms (SNPs) are tested simultaneously in thousands of cases and controls for association with a human complex disease - have revolutionized the search for genetic basis of these diseases [1]. The success of GWAS can be summarized with the published 600 genomewide association studies covering 150 distinct diseases and traits, explaining 800 SNP-trait associations (P<5×10^−8^). These studies not only identified novel common genetic risk factors, but also confirmed the importance of previously identified genetic variants. However, in a typical GWAS, only a minority of DNA sequence variations that modulate disease susceptibility and their neighboring genes with the strongest evidence of association is explained. Whereas, in this “most-significant SNPs/genes” approach, genetic variants that confer a small disease risk but are of potential biological importance are likely to be missed. Hence, it is recognized that GWAS data is undermined in most cases and concentrating on a few SNPs and/or genes with the strongest evidence of disease association is not enough to exploit underlying physiological processes and disease mechanisms [2]. For instance, PPARG variants are known to be associated with type 2 diabetes (T2D) [3]. Whereas, this true association is missed by the four out of five GWA studies designed to replicate the initial finding, due to its modest effect on disease susceptibility (odds ratio 1.2) [4], [5]. A similar situation was recently observed regarding the association of IL7R variants with multiple sclerosis [5]. Especially in complex diseases, which are intrinsicly multifactorial, rather than identifying single genes, the identification of affected pathways would shed light into understanding of disease development mechanism.

Pathway-based approaches thought to complement the most-significant SNPs/genes approach and provide additional insights into interpretation of GWAS data on complex diseases [2], [5], [6], [7]. These pathway-based GWASs are based on the hypothesis that multiple genes in the same biological pathway contribute to disease etiology, wheras common variations in each of these genes make mild contributions to disease risk. The use of prior knowledge in the form of pathway databases is demonstrated in GWAS of diseases such as Parkinson's disease, age-related macular degeneration, bipolar disorder, rheumatoid arthritis, and Crohn's disease [8], [9], [10], [11], [12]. While the concept of pathway analysis for GWAS is attractive, it is restricted by our limited knowledge of cellular processes.

On the other hand, a limited number of studies have attempted to incorporate network-based analysis to interrelate positional candidate genes from disease loci and/or to prioritize candidate loci in genetic studies [13], [14], [15], [16]. However, these studies either do not use actual genetic (genotypic) data or are applied to model organisms. To the best of our knowledge, the only study to date that uses both a protein interaction network and pathway analysis to reveal significant disease related genes and pathways in genetic association studies is conducted by Baranzini et al. [5] on Multiple Sclerosis. Since this study is gene centered, it is possible that true associations with markers that lie in large intergenic regions were neglected and the analysis is limited to the known functional properties of genes. Additionally, to improve the power in GWAS, Roeder et al. developed a method to incorporate linkage data to weight the association P values [17]; and a weighted multiple testing procedure that facilitates the input of prior information in the form of groupings of tests [18]. In this study, they have shown that the grouped-weighting of prior information often leads to an increase in power even if many of the groupings are not correlated with the signal [18].

To further reduce the number of selected SNPs after a GWAS, here we hypothesize that researchers need to integrate information from various biological databases, where biologically significant SNPs, such as those occurring in functional genomic regions such as protein-coding or regulatory regions; or those located in genes related to the phenotype are given higher priority. In this light, we present a pathway and network oriented GWAS analysis (PANOGA) that challenges to identify individually modest genetic effects by combining nominally significant evidence of genetic association with current knowledge of biochemical pathways, protein-protein interaction networks, and functional and genetic information of selected SNPs. Starting with GWAS data, our proposed methodology assigns genes into functionally important Kyoto Encyclopedia of Genes and Genomes (KEGG) pathways (http://www.genome.ad.jp/kegg/pathway.html). In addition to the network and pathway-based analysis, PANOGA incorporates a regional score, which integrates functional properties of a SNP that is found to be important in GWAS.

We applied our methodology on a GWAS of Rheumatoid Arthritis (RA); and identified both previously found RA related KEGG pathways and additional pathways. We compared our findings with the known disease genes collected from the OMIM database (http://www.ncbi.nlm.nih.gov/Omim), NCBI PubMed module RA-specific drug target genes obtained from Pharmaccogenomics Knowledge Base website (http://www.pharmgkb.org/index.jsp); and with their KEGG functional enrichments. Our analysis highlights the importance of particular genes that have already been identified as significant in the pathogenesis of RA, gives more insights into their potential role considering their biological pathways, and shed light into their ability to affect neighboring pathways (presented as functional annotation network). The strength of our methodology stems from its multidimensional perspective, where we combine evidence from the following 4 resources: i) Genetic association information obtained through GWAS, ii) SNP functional information, iii) Protein-protein interaction data, iv) Biochemical pathways. In summary, we showed that using our technique, a GWAS can be mined further to identify novel genes and pathways that are associated with a specific human complex disease. In the following sections we present our findings and identify areas for further research. As more biological knowledge and genomic data become publicly available, we believe that such methodological developments will better dissect the genetic architecture of human complex diseases.

## Methods

**Figure 1:**
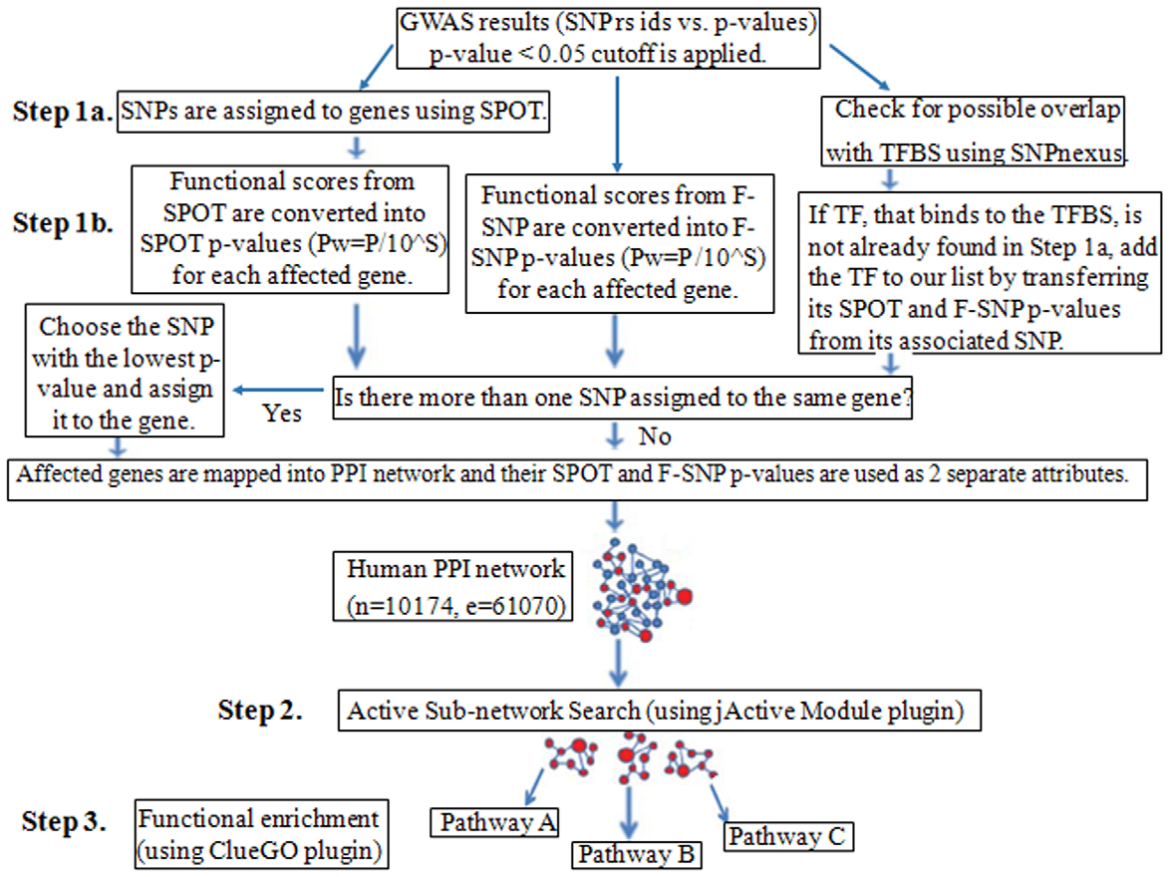
Outline of our assessment process. In Step 1, a gene-wise Pw-value for association with disease was computed by integrating functional information. In Step 2, significant Pw-values were loaded as two separate attributes of the genes in a PPI network and visualized using Cytoscape [23]. At this step, active sub-networks of interacting gene products that were also associated with the disease, are identified using jActive Modules plugin [24]. In Step 3, genes in an identified active sub-network were tested whether they are part of functionally important KEGG pathways.

Starting with a list of SNPs, found to be associated with disease in GWAS, we propose a novel methodology to devise the list of genes included in a functionally important KEGG pathway. In our study, GWAS results are used in the form of SNP rs ids vs. p-values, where the p-values refer to the genotypic p-values of association for each tested SNP. We only focused on SNPs with nominal evidence of association (P<0.05) in a GWAS, following the study in [5]. Our system proceeds in three main steps as outlined in Figure 1. In Step 1a, SNPs are assigned to genes based on SNP/gene transcript functional properties. In order to incorporate functional information, a SPOT [19] and F-SNP [20], [21] Pw-values are assigned to each gene as two separate attributes in Step 1b. This step also checks whether the input SNPs overlap with known Transcription Factor Binding Sites (TFBS) at TRANSFAC [22]. These functional properties are assigned as gene attributes to a human protein protein interaction map in Step 2. Lastly, Step 3 conducts functional enrichment and assigns genes into functionally relevant KEGG pathways. We further describe each step below.

### Step 1a: Assigning SNPs to genes

It is hypothesized that meaningful combination of genes harboring markers with only modest evidence of association can be identified if they belong to the same biological pathway or mechanism [5]. Therefore, the gene and pathway-based association analysis allows us to gain insight into the functional basis of the association and facilitates to unravel the mechanisms of complex diseases. However, a SNP may be associated with many genes, i.e. it can be located in a gene with several known transcripts due to alternative splicing, or in one gene and very close to another gene, or at the intersection of different genes on different strands and hence a SNP may have different functional consequences on each transcript. To assign SNPs into genes by considering all known SNP/gene transcript associations, our methodology uses SPOT program [19] which selects the gene with the highest priority. To generate those SNP/gene transcript associations, SPOT program utilizes information from the PolyPhen method of predicting the effect of an amino acid substitution on the properties of the protein product. Those effects can be directly detected from DNA and RNA sequences, like nonsense and missense amino acid substitutions, untranslated regions, coding regions, and frameshifts. Hence, by prioritizing all known SNP/gene transcript consequences, propitious association signals found in GWAS, are not lost at the SNP to gene transition step.

### Step 1b: Regional Score Calculations for Genes

In this step, our methodology combines functional, genomic information of a SNP with P-value of a SNP from a statistical test for genetic association, and then transfers this weighted P-value (Pw-value) to the SNP's associated gene. Among many different web tools dealing with SNP biological properties, we have decided to combine the scores of SPOT [19] and F-SNP [20] servers. A comprehensive comparison of those meta-tools can be found elsewhere in literature, but notably in a review paper [25].

SPOT score [19] takes into account SNP/gene transcript functional properties (including nonsense, frameshift, missense and 5′ and 3′-UTR designations), impact of an amino acid substitution on the properties of the protein product from PolyPhen server [26], [27], evolutionary conserved regions from ECRbase [28], all possible LD proxies - SNPs with r^2^ over a predefined threshold in a specific HapMap sample [29]. On the other hand, F-SNP score (FS score) incorporates: functional effects of SNPs, predicted at the splicing, transcriptional, translational and post-translational level [20]. The details of the data sources used in our regional score can be found in Table 1.

**Table 1:** Description of data sources used in our regional score.

Functional Category	Tool	Description	Meta-tool
Protein Coding	LS-SNP, SNPs3D, SIFT, SNPeffect	SNP annotation tool, Impact of nsSNPs on protein function, Prediction of amino acid substitution effects, SNP annotation with human disease	F-SNP
Protein Coding	PolyPhen	Prediction of amino acid substitution effects	SPOT,F-SNP
Protein Coding, Splicing Regulation, Transcriptional Regulation	Ensembl	Extensive genomic database including SNPs and gene transcripts	F-SNP
Splicing Regulation	ESEfinder, ESRSearch, PESX, RescueESE	Exonic splice sites, Exonic-splicing regulatory (ESR) sequences, Exon splicing enhancers/silencers, Exonic splice sites	F-SNP
Transcriptional Regulation	ConsiteTFSearch	Conserved transcription factor binding sites,Transcription factor binding sites	F-SNP
Transcriptional Regulation	SNPnexus	Conserved transcription factor binding sites	SNPnexus
Transcriptional Regulation, Conserved Region	GoldenPath	MicroRNA, cpgIslands, evolutionary conserved regions	F-SNP
Conserved Region	ECRBase	Evolutionary conserved regions	SPOT
Post-translation	KinasePhos, OGPET, Sulfinator	Phosphorylation sites, Prediction of O-glycosylation sites in proteins, Tyrosine sulfination sites	F-SNP
Genomic Coordinates	dbSNP	General SNP/gene transcript properties	SPOT
Genomic Coordinates	UCSC	Extensive genomic database including SNPs and gene transcripts	F-SNP
LD estimation	HapMap,Haploview	Dense genotyping on multiple populations, useful for LD estimatesEstimation of r^2^ LD coefficients for each population	SPOT

To combine biological information with evidence for genetic association, the following scoring scheme is proposed in [30]. In [30], firstly, a non-negative prioritization score (PS) was specified for each SNP and then, the weighted P-value P_w_ is defined by P_w_ = P/10^PS^ [17], [30], where P denotes GWAS p-value for a particular SNP. In this scheme, smaller values of P_w_ indicate higher priority. Following this convention, we have calculated SPOT Pw-value using SPOT prioritization score and F-SNP Pw-value using F-SNP prioritization score. Since SNPs are associated with genes in Step 1a of our method, these two weighted p-values (Pw-values) are automatically transferred into the SNP's associated gene as two separate attributes. Hence each gene has a SPOT Pw-value and a F-SNP Pw-value for association with RA (gene-wise Pw-values). If more than one SNP is assigned to the same gene in Step 1a, the SNP with the lowest weighted p-value (Pw) is chosen and assigned to the gene. In other words, the SPOT Pw-value of a gene is calculated as the lowest SPOT Pw-value of the SNP that is assigned to that particular gene among all the SPOT Pw-values of the SNPs assigned to the same gene. Same is true for F-SNP Pw-value.

Lastly, SNPnexus [31] checks for possible overlap of a SNP with conserved TFBSs from TRANSFAC Matrix Database (v.7.0, [22]) and returns the related TF name. If this TF is not already found in Step 1a, this TF is added to our list by transferring its SPOT and F-SNP Pw-values from its associated SNP.

### Step 2: Active sub-network searches

By using the regional scores, calculated in the previous step for the genes, this step aims to find out active sub-networks in the human PPI network. Firstly, a human PPI network was imported into Cytoscape [23]. Secondly, regional scores (SPOT and F-SNP Pw-values) were loaded as attributes of the genes in this network. Lastly, active sub-networks of interacting gene products that were possibly associated with the disease are identified using jActive Modules [24], [32] in a formal way. Basicly, jActive Modules [24] is a Cytoscape plugin to identify active sub-networks via incorporating both the topological properties of a PPI network and the attributes of the nodes (proteins). In this approach, firstly the attributes (SPOT and F-SNP Pw-values) are mapped into biological networks, secondly a statistical measure (as explained below) is used to score sub-networks based on the attributes, and finally a search algorithm is used to find active sub-networks with high score.

Biologically speaking, an active sub-network (statistically significant module) is a sub-network in our PPI network that the protein products of this set of genes – probably associated to the disease- also physically interact, thus raises the possibility that they belong to the same pathway or biological process. To rate the biological activity of a particular sub-network, jActive Modules starts by assessing the significance of differential association with disease for each gene (by comparing the gene-wise Pw-values of association with the disease). In this procedure, jActive Module samples p-values from the distribution of p-values loaded into Cytoscape, and not from a normal uniform distribution. Then, a network is generated from each node by systematically adding one neighbor at a time. The aggregate z-score (S) of an entire sub-network, consisting of k genes is calculated via summing the scores of all genes z_i_ in the sub-network and then dividing by the square-root of k. To extend the z-score over multiple conditions (attributes), jActive Module sorts z-scores for each attribute, adjusts for rank, maximum score is corrected using the background score distribution [24]. The scoring system of jActive Modules ensures that the expected mean and variance of the subgraph scores are independent of subgraph size [24]. jActive Modules plugin also corrects for the fact that a bigger sub-network is more likely to contain nodes with significant p-values by random chance [24]. When S stops to increase, the sub-network stops growing and is reported as a module. Next, the test statistic (S) is compared with an appropriate background distribution to properly capture the connection between network topology and association with disease. As a background distribution, we used the scores of sub-networks randomly selected from the entire human PPI network, as provided by jActive Modules. In order to make the background distribution independent of the module size, jActive Modules creates a background distribution by scoring 10,000 random sub-networks of each size in a Monte Carlo procedure. In our study, modules with S>3 were reported as significant (active sub-network), as stated in the original publication. The sub-network with the highest score is selected for further functional enrichment.

### Step 3: Functional Enrichment of the Sub-networks

Next step following the identification of sub-networks is to evaluate whether these sub-networks were biologically meaningful. Our methodology has a functional enrichment component that computes the proportion of the genes in an identified sub-network that are also found in a specific human biochemical pathway, compared to the overall proportion of genes described for that pathway. For this purpose, ClueGO plugin of Cytoscape [33] is utilized in this step. ClueGO is an open-source Java tool that extracts the non-redundant biological information for groups of genes using GO, KEGG and BioCarta ontologies [33]. Unlike other functional enrichment analysis tools [34], [35], [36], [37], [38] that present their results as long lists or complex hierarchical trees; ClueGO facilitates the biological interpratation via visualizing functionally grouped terms in the form of networks and charts [33]. To link the terms in the network, ClueGO uses kappa statistics, in a similar way as described in [35]. Among different ontologies, since KEGG database primarily categorizes genes into bona-fide biological pathways; and since biological interpretation of pathways is more straightforward compared to GO terms, we report only our functional enrichment results using KEGG pathways. To determine the statistical significance of an enrichment of the identified sub-network, two-sided (Enrichment/Depletion) test based on the hypergeometric distribution is used in our methodology. To correct the *P*-values for multiple testing, Bonferroni correction method is applied.

### Experiments

Rheumatoid Arthritis (RA, OMIM # 180300) is a systemic inflammatory disease, primarily affecting synovial joints. As reported at the 2008 American College of Rheumatology meeting, about 1% of the world's population is afflicted by RA and women affected three times more often than men. Disease onset is most frequent between the ages of 40 and 50, but people of any age can be affected. While the earlier stages of the disease appear a disabling and painful condition, in the later stages it can lead to substantial loss of functioning and mobility.

Being a complex disease, the etiology of RA depends on a combination of multiple genetic and environmental conditions, involving a yet unknown number of genes. The heritability of this disease is estimated as ∼50% based on family studies, including twin studies [39], [40]. In GWASs among RA patients of European ancestry, multiple risk alleles have been identified in the major histocompatibility complex (MHC) region, and 25 RA risk alleles have been confirmed in 23 non-MHC loci [41], [42], [43], [44], [45], [46], [47], [48], [49], [50], [51]. These variants explain about 23% of the genetic burden of RA [41], indicating that additional variations remain to be discovered to explain the polygenic etiology of RA.

### Genetic Association Data of Rheumatoid Arthritis

We have applied our methodology on Wellcome Trust Case Control Consortium (WTCCC) Rheumatoid Arthritis (RA) dataset, in which 500,475 SNPs were tested on 5003 samples (1999 cases and 3004 controls) using Affymetrix GeneChip Human Mapping 500 K Array Set. SNP data and the genotypic P-values of association for each tested SNP were downloaded from the WTCCC project webpage (www.wtccc.org.uk). In total, 25,027 SNPs were included from WTCCC dataset, showing nominal evidence of association (P<0.05).

### Protein-protein interaction (PPI) data

A human protein-protein interaction (PPI) data was obtained from the supplementary material of Goh et al. 's study [52]. This dataset is composed of two high quality systematic yeast two-hybrid experiments [53], [54] and PPIs obtained from literature by manual curation [53]. The integrated set of PPIs contains 61,070 interactions between 10,174 genes (22,052 non-self-interacting, non-redundant interactions).

## Results

Starting with 25,176 SNPs, that are found to be significant in a GWAS (WTCCC RA dataset), PANOGA was performed to identify RA related genes and functionally important KEGG pathways. These SNPs were assigned into 4,029 genes using SPOT webserver [19] by considering all known SNP/gene transcript associations. As the possible overlap of a SNP with conserved TFBSs was considered, by using SNPnexus program [31], we incorporated 65 more proteins (TFs) that bind to the TFBS, that an RA associated SNP resides in. In order to incorporate functional information (regional score) to these genes, SPOT and F-SNP Pw-values were calculated as mentioned in the methods section. Following these calculations, network oriented steps of the PANOGA was realized using Cytoscape [23]. SPOT and F-SNP Pw-values were used as attributes of the nodes (4094 genes) in the curated PPI network. We next searched for active sub-networks using the Cytoscape plugin jActive Modules. This plugin combines the network topology with attributes (SPOT and F-SNP Pw-values in our case) of each gene to extract potentially meaningful sub-networks. The higher the assigned aggregate z-score of a sub-network is, biologically more active the sub-network is. As in the original publication of jActive Modules [24], sub-networks with a score S>3 (3 SD above the mean of randomized scores) were considered significant. Hence, our results with scores around 16 showed that this sub-network is statistically significant. But the involvement of the genes in this network with RA is further investigated through comparison with existing RA related information in databases.

### Significant sub-networks for RA

**Figure 2:**
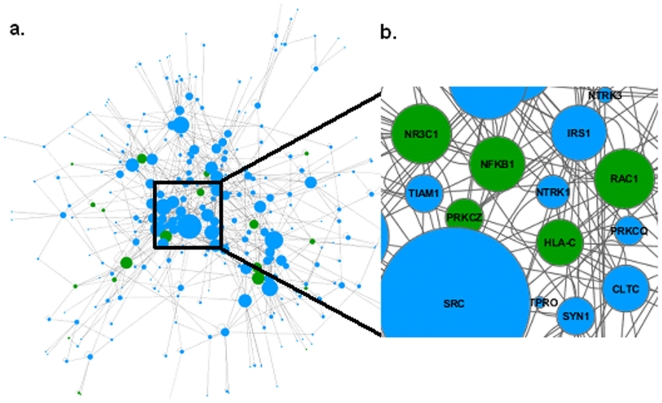
The highest scoring sub-network. **a.** This sub-network is composed of 275 nodes and 778 edges (as found in Step 2 of PANOGA). Node size is shown as proportional to the degree of a node. **b.** Zoomed in view of the highest scoring sub-network. 20 genes known in literature as associated with RA are shown in green. Blue denotes the genes in our highest scoring sub-network that cannot be associated with RA in literature.

Using both GWAS p-values and regional score, we identified 5 significant sub-networks on the basis of their aggregate degree of genetic association with RA. Due to the nature of the search algorithm, several of these sub-networks overlap extensively in their component genes. Thus, to describe a sub-network representative of association with RA, we selected the one with the highest score. This selected active sub-network is composed of 275 genes (our gene set) and 778 edges, as shown in Figure 2a. Associations between 20 genes from this sub-network (XCL1, VCAM1, TRPV1, TRPC1, SPP1, RUNX1, RAC1, PRKCZ, NR3C1, NFKB1, MAP2K4, JUN, ITGB1, ITGAV, HMGB1, HLA-DMB, HLA-C, ERBB2, EPAS1, CCL21) and RA were verified by literature retrieved from the NCBI PubMed module and OMIM, as shown in Figure 2b.

**Figure 3:**
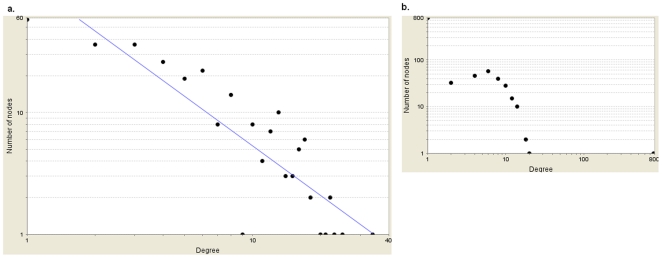
Node degree distributions of our highest scoring sub-network vs. random network. **a.** Our sub-network follows a power-law (P(k) = ax^−γ^, a = 120.03, γ = 1.353, R^2^ = 0.773, Correlation = 0.891 in log log scale), showing that our network displays scale-free properties, as expected from a biological network. **b.** The random network is obtained via randomization of our highest scoring sub-network using Erdos-Renyi algorithm.

Next, we checked the topological parameters of this network. The distribution of the number of links per node (degree distribution, P(k)) is an important measure for a network to decide if it is a random, scale-free or hierarchical network. As shown in Figure 3a, the degree distribution of our highest scoring sub-network follows a power-law distribution (P(k) = ax^−γ^, a = 120.03, γ = 1.353, R^2^ = 0.773, Correlation = 0.891 in log log scale) and hence it is scale-free, as expected from a biological network [55], [56], [57], [58]. The unusual properties of scale-free networks are valid only for γ<3 and the smaller the value of γ, the more important the role of the hubs is in the network [59]. Similar to the degree distribution of the main PPI network (γ = 1.617), the degree distribution of other top 5-scored sub-networks follows a power-law distribution (γ = 1.418, 1.365, 1.406, 1.330). We also randomized our highest scoring sub-network using Erdos-Renyi algorithm and observed that its node degree distribution follows a Poisson distribution as expected from a random network (Figure 3b).

### Functionally important KEGG pathways for RA

As a result of the functional enrichment step (Step 3) of our methodology, we identified 87 KEGG pathway terms. In Table 2, we represent 20 most significant pathways (determined by their p-values), which are mostly related to immunity and inflammation, cell adhesion and cancers. Most of these pathways (Chemokine signaling, Neurotrophin signaling, Pathways in cancer, Leukocyte transendothelial migration, T cell receptor signaling, Toll-like receptor signaling, Allograft rejection, MAPK signaling, Apoptosis, Jak-STAT signaling) have been previously found to be associated with RA experimentally. In Table 2, we formatted the pathways and genes in italic, bold, both italic and bold, respectively, if they are computationally found only, experimentally found only, or found both experimentally and computationally. For example, Toll-like receptor (TLR) signaling pathway term was formatted in both italic and bold since other computational methods identified this term and it is also experimentally known to play an important role in the development and progress of RA. Among the most significant pathways identified by our methodology are Focal Adhesion and Cell Adhesion Molecules (CAM) pathways. These pathways are experimentally shown to play a critical role in cellular processes such as osteoclass pathology and angiogenesis, which are known to be important for RA [60].

**Table 2:** Overrepresented KEGG Pathways found in the highest scoring sub-network as associated with RA.

KEGG Term	Num. of Genes Found	Asso-ciated Genes (%)	Term Pvalue Corr. w/Bonfer.	Associated Genes Found
*Focal adhesion*	30	14,9	9,33E-11	ACTB, ACTG1, AKT1, COL4A4, CRKL, CTNNB1, EGF, EGFR, FLNA, FLNB, FLT4, FYN, GRLF1, ITGA5, ***ITGB1***, *ITGB3*, *ITGB5*, MAP2K1, PAK4, PIK3R2, PTK2, **RAC1**, RHOA, *SHC3*, SRC, VASP, VAV1, VAV3, VTN, ZYX
*ErbB signaling pathway*	20	22,9	2,13E-10	AKT1, *CAMK2D, CAMK2G*, CBL, CRKL, EGF, EGFR, ERBB3, ERBB4, HBEGF, KRAS, MAP2K1, NCK2, NRG1, PAK4, PIK3R2, PTK2, *SHC3*, SRC, STAT5A
*Tight junction*	22	16,4	1,80E-08	ACTG1, ACTN2, CASK, CTNNB1, EPB41L1, EPB41L2, EPB41L3, GNAI1, INADL, KRAS, LLGL1, MAGI1, MAGI3, PARD3, PRKCE, PRKCI, **PRKCQ**, **PRKCZ**, RHOA, SPTAN1, SRC, TJP1
**Chemokine signaling pathway**	26	13,7	2,31E-08	ADCY2, ADCY5, *ADCY8*, AKT1, *CHUK*, CRKL, DOCK2, ELMO1, FGR, GNG2, IKBKB, KRAS, MAP2K1, NCF1, PARD3, PIK3R2, PRKCZ, PTK2, PTK2B, RAC1, RHOA, SHC3, STAT3, TIAM1, VAV1, VAV3
*Adherens junction*	17	22,6	1,16E-07	ACTB, BAIAP2, CREBBP, CTNNB1, *EP300*, FYN, PARD3, PTPRF, *PTPRM*, RHOA, SMAD2, SMAD4, SORBS1, SRC, TCF7L2, TGFBR1, TJP1
Bacterial invasion of epithelial cells	15	20,5	1,57E-07	ACTB, ACTG1, CBL, CLTC, CTNNB1, CTTN, DNM3, ELMO1, ***ITGB1***, *PIK3R1*, PTK2, **RAC1**, RHOA, SRC, WASL
**Neurotrophin signaling pathway**	20	15,8	2,36E-07	ARHGDIB, CALM1, CALM3, *CAMK2D*, IKBKB, IRS1, JUN, KRAS, MAPK10, MAPK3, NFKB1, NTRK1, NTRK3, PLCG1, RAC1, RHOA, RPS6KA1, TP73, YWHAE, YWHAH
*Long-term potentiation*	15	21,4	3,67E-07	*ADCY8*, CALM1, CALM3, *CAMK2D*, *EP300*, GRIA1, GRIN1, GRIN2B, GRM5, *ITPR1*, ITPR3, KRAS, MAPK3, PPP1CB, RPS6KA1
**Pathways in cancer**	32	9,7	1,12E-06	*CASP8*, CBL, *CHUK*, COL4A4, CTNNB1, *EP300*, **EPAS1**, ERBB2, FOXO1, FZD4, IKBKB, ITGAV, ***ITGB1***, JUN, KIT, KRAS, MAPK10, MAPK3, NFKB1, NTRK1, PIAS1, PIAS2, PLCG1, PTK2, RAC1, RHOA, RUNX1, SMAD4, STAT1, STAT5A, TCF7L2, TPM3
*Chronic myeloid leukemia*	14	19,1	1,44E-06	CBL, CRK, CRKL, *HRAS*, IKBKB, *MAPK3*, ***NFKB1***, PIK3R2, SHC1, SMAD3, SMAD4, SOS1, STAT5B, TGFBR1
*Cell adhesion molecules (CAMs)*	18	13,2	1,42E-05	CD226, **CD28**, *CD4*, CDH2, HLA-B, **HLA-C, ** ***HLA-DMB***, *HLA-DPA1*, *HLA-DQA2*, *HLA-DRA*, ***ITGB1***, L1CAM, NCAM1, *NLGN1*, ***PTPRC***, PTPRF, *PTPRM*, **SDC3**
***Leukocyte transendothelial migration***	17	11	1,72E-05	ACTG1, ACTN2, CTNNB1, EZR, GNAI1, GRLF1, ***ITGB1***, NCF1, PLCG1, PTK2, PTK2B, **RAC1**, RHOA, *TXK*, VAV1, VAV3, VCAM1
***T cell receptor signaling pathway***	16	14,8	2,70E-05	CBL, *CD247*, **CD28**, *CD4*, *CHUK*, FYN, *HRAS*, IKBKB, LCK, MAP2K1, NCK2, PLCG1, **PRKCQ**, ***PTPRC***, RHOA, VAV3
***Toll-like receptor signaling pathway***	13	12,7	1,97E-03	*CASP8*, *CHUK*, *IFNAR1*, IFNAR2, IKBKB, JUN, MAP2K4, MAPK10, MAPK3, ***NFKB1***, **RAC1**, SPP1, STAT1
*Antigen processing and presentation*	11	13,9	2,08E-03	CALR, CANX, HLA-B, **HLA-C, ** ***HLA-DMB***, *HLA-DRA*, HLA-F, **HLA-G**, HSPA1L, **TAP1**, **TAP2**
**Allograft rejection**	8	20	2,16E-03	**CD28**, HLA-B, **HLA-C**, ***HLA-DMB***, *HLA-DPA1*, *HLA-DQA2*, *HLA-DRA*, IL12A
***MAPK signaling pathway***	20	7,4	6,13E-03	CACNA1A, *CHUK*, CRKL, DAXX, EGF, FLNA, FLNB, FOS, *HRAS*, HSPA1L, JUN, MAPK10, *MAPK3*, MAPK8, NF1, **RAC1**, RPS6KA1, RRAS2, SOS1, TGFBR1
*Type I diabetes mellitus*	8	17,3	6,24E-03	**CD28**, HLA-B**, HLA-C**, ***HLA-DMB***, *HLA-DPA1*, *HLA-DQA2*, *HLA-DRA*, IL12A
***Apoptosis***	11	12,5	6,84E-03	CAPN1, *CASP10*, *CASP8*, *CHUK*, *CSF2RB*, FADD, IKBKB, IRAK1, *IRAK4*, PRKAR2A, PRKAR2B
***Jak-STAT signaling pathway***	15	9,6	7,41E-03	CBL, CREBBP, *CSF2RB*, *EP300*, *IFNAR1*, IFNAR2, IL12A, ***IL2RA***, ***IL2RB***, JAK1, LIFR, SOCS5, STAT1, STAT3, STAT5A

Bold formatting denotes experimentally verified RA associated genes and pathways, italic formatting denotes computationally found, RA associated genes and pathways, bold and italic formatting denotes both experimental and computational verification regarding susceptibility to RA.

We compared our findings with previously found RA related KEGG pathways and with the genes found from those pathways. Wu et al. [61], created a comprehensive molecular interaction map for RA by combining the molecules and pathways found to be associated with RA based on merging all available papers related to high throughput experiments on RA. Following a procedure as in [62], they have decomposed their network into 11 modules using the Cytoscape plugin BiNoM [63]. DAVID [35] pathway analysis on their largest module with 292 nodes for 104 proteins and 334 edges returned 26 different KEGG pathways. In summary, this module contains 43 proteins from the MAPK signaling pathway, 36 proteins from focal adhesion, 23 proteins from the ErbB signaling pathway, and some cancer associated pathways such as leukemia, prostate cancer and colorectal cancer.

In another study by Martin et al., 2010, the genomic regions showing low-significance associations in previous GWAS of RA (WTCCC and NARAC datasets) were further explored. Using Prioritizer software [13], they have prioritised genes from similar pathways but located in different regions. This tool searches for those genes belonging to the same biological pathways or related biological pathways, based on the assumption that true disease-causing genes are functionally related. Prioritizer software uses a Bayesian approach to reconstruct a functional gene network based on known functional interactions from several databases such as the KEGG. Martin et al., 2010 reported 18 overrepresented KEGG pathways; in which Jak-STAT signaling pathway, Glioma, Calcium signaling pathway, Long-term potentiation, Apoptosis had the top 5 scores.

Baranzini et al., 2009 conducted a pathway-oriented analysis on WTCCC GWAS data for RA and another GWAS data by Plenge and collaborators. 9 KEGG pathways were identified in this study including Cell adhesion molecules (CAMs), Antigen processing and presentation, Type I diabetes mellitus. Lastly, the screening approach developed by Zhang et al., 2010 to further analyze GWAS data considers all SNPs with nominal evidence of Bayesian association, structural and functional similarities of corresponding genes. Responsible pathways identified in their study include Jak-STAT signaling pathways, cell adhesion molecules, and MAPK signaling pathways.

Comparative results with these three studies are shown in Table 3 in terms of number of genes found in commonly identified KEGG pathways. While most of these associations are computational predictions only, the functional relations of five of these pathways (Jak-STAT signalling, apoptosis, T cell receptor signalling, leukocyte transendothelial migration and cytokine-cytokine receptor interaction) with RA pathogenesis are known [42], [46]. Also, the effect of Toll-like receptor (TLR) signaling pathway and MAPK signaling pathway on RA is known. Here it is important to note that these associations are obtained by different methods on different datasets. For example, while Wu et al. utilizes text-mining [61], Martin et al. mines GWAS data from WTCCC and NARAC studies (including variations on more cases and controls) [64], and Zhang et al. applies their methodology on GAW16 (Genetic Analysis Workshop) data [65]. PANOGA identifies previously found KEGG pathway terms with high statistical significance (terms shown in italic format for former computational identification, in italic and bold for both computational and experimental identification).

**Table 3:** Comparison of found KEGG pathways with previous studies in terms of number of genes associated within each KEGG term.

KEGG Term	Number of Genes Found						Term Pvalue Corrected Bonfer-roni
	Baranzini et.al.	Martin et.al.	Wu et.al	Zhang et.al.	PANOGA (only GWAS p-values)	PANOGA (w/2 attributes SPOT Pw and F-SNP Pw)	
*Focal adhesion*	0	0	36	32	22	30	9,33E-11
*ErbB signaling pathway*	0	0	23	0	18	20	2,13E-10
*Tight junction*	0	0	0	5	20	22	1,80E-008
**Chemokine signaling pathway**	0	0	0	0	24	26	2,31E-08
*Adherens junction*	0	0	0	18	16	17	1,16E-07
Bacterial invasion of epithelial cells	0	0	0	0	15	16	1,57E-007
**Neurotrophin signaling pathway**	0	0	0	0	20	20	2,36E-07
*Long-term potentiation*	0	22	0	7	14	15	3,67E-07
**Pathways in cancer**	0	0	0	0	29	32	1,12E-06
*Chronic myeloid leukemia*	4	0	21	18	10	14	1,44E-06
*Cell adhesion molecules (CAMs)*	8	26	0	10	12	18	1,42E-05
***Leukocyte transendothelial migration***	0	24	14	0	17	17	1,72E-05
***T cell receptor signaling pathway***	4	21	16	16	13	16	2,70E-05
***Toll-like receptor signaling pathway***	0	0	22	6	7	13	1,97E-03
*Antigen processing and presentation*	6	0	0	3	11	11	2,08E-03
Allograft rejection	0	0	0	0	8	8	2,16E-03
***MAPK signaling pathway***	0	0	43	34	16	20	6,13E-03
*Type I diabetes mellitus*	5	0	0	1	8	8	6,24E-03
*Apoptosis*	0	18	12	11	6	11	6,84E-03
***Jak-STAT signaling pathway***	0	25	0	16	13	15	7,41E-03
*Prostate cancer*	0	0	22	0	10	11	5,04E-02
*Calcium signaling pathway*	0	35	0	4	15	16	1,63E-01
*VEGF signaling pathway*	3	0	15	13	8	9	2,71E-01
Total	30	171	224	194	332	385	

Italic formatting denotes computationally found pathways, bold formatting denotes experimentally verified RA associated pathways, bold and italic formatting denotes both experimental and computational verification.

From those previously identified pathways, we identified additional genes associated with RA within some of these pathways (e.g. Antigen processing and presentation, Tight junction). Importantly, within these pathways, the associations between some of these additionally found genes, such as HLA-C, HLA-G, PRKCQ, PRKCZ, TAP1, TAP2 (formatted in bold in Table 2) and RA were also verified by either OMIM database or by literature retrieved from the NCBI PubMed module.

Different from previous studies, we also identified Chemokine signaling, Neurotrophin signaling, Pathways in Cancer, Allograft rejection pathways as significant for RA. While the significance of these pathways in relation to RA were not thoroughly discussed in literature, the KEGG functional enrichment of RA-specific drug target genes, included these terms (whole list of drug target genes for RA, downloaded from Pharmaccogenomics Knowledge Base website and whole list of the KEGG functional enrichment of these genes can be found in Supplementary Tables S1 and S2, respectively). In this database, 83 genes are associated with drugs that are used to treat RA. Furthermore, within these pathways, the associations between some of the genes, such as EPAS1, CD28, HLA-C (formatted in bold in Table 2) and RA were verified by either OMIM database or by literature retrieved from the NCBI PubMed module.

In order to assess the contribution of the found pathways and associated genes to disease mechanism, we also searched all identified genes from all found pathways in the Pharmaccogenomics Knowledge Base website. When we filtered SNPs based on their significance in GWAS (p-value <0.05 cutoff is applied) and assigned into genes, 14 out of 85 drug target genes were found. Whereas, via considering all the genes in the found KEGG pathways, we identified 25 out of 85 drug target genes associated with RA (listed in Supplementary Table S3). Hence, we showed that incorporating pathway knowledge on top of GWASs provides additional insights into the pathogenesis of RA.

To emphasize the effect of the regional score in PANOGA, we have applied our analysis on 4,094 genes firstly by using only GWAS p-values, secondly by using both SPOT and F-SNP Pw-values as attributes. As can be seen in Table 3, (PANOGA (w/ regional scores) column vs. PANOGA (only GWAS pvalues) column), incorporating functional information of a SNP increases the number of genes identified as associated with RA; and hence increases the significance of the identified KEGG pathway term.

### Functionally grouped annotation network of RA

**Figure 4:**
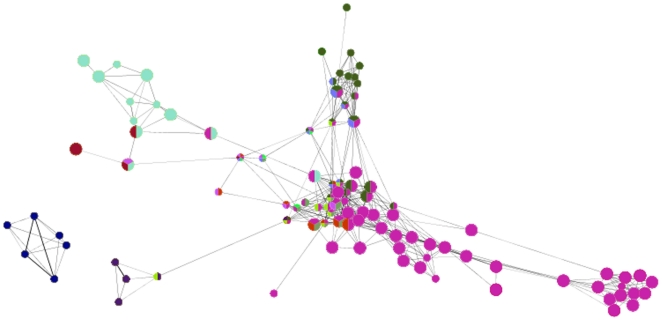
Functionally grouped annotation network of our highest scoring sub-network. The relationships between the KEGG terms (nodes) were based on the similarity of their associated genes. The size of the nodes reflected the statistical significance of the terms (term p-values corrected with Bonferroni). Edges represent the existence of shared genes. The thickness of the edges is proportional to the number of genes shared and calculated using kappa statistics, in a similar way as described in [35]. The grouped terms (according to their kappa scores) were shown in same color.

**Figure 5:**
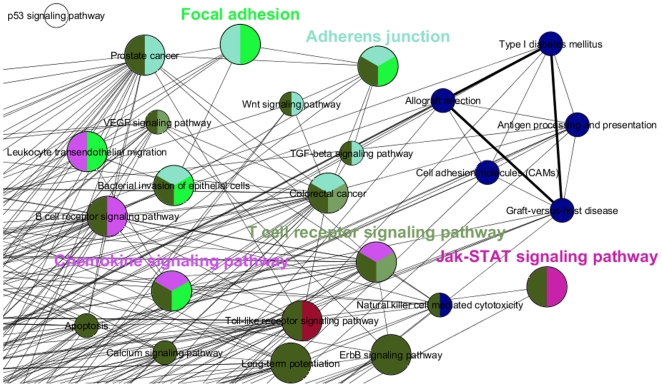
Zoomed in view of the entire functional annotation network. The most significant pathway term of the group with the lowest term p-value (the group leading term) was shown in bold using the group specific color.

The diversity and complexity of the identified KEGG pathways involved in one sub-network confirms that RA is a complex systemic disease. Since a gene can be present in multiple pathways, we would like to show the pathway relationship, based on whether the pathways are sharing same genes. Hence, we generated a functional annotation network from the found KEGG pathways using ClueGO plugin [33]. While the nodes in a functionally grouped network in Figure 4 denoted the found KEGG terms associated to RA, the edges were drawn based on the existence of shared genes using kappa statistics, in a similar way as described in [35]. 87 pathway terms that were found to be RA associated in our analysis were clustered into 9 groups, as can be seen in Figure 4 (according to their kappa scores) and the pathways in the same group were shown in same color. ClueGO also assigns the most significant pathway terms with the lowest term p-value (corrected with Bonferroni) as group leading terms. For our functional annotation network, Focal adhesion, Adherens junction, Chemokine signaling pathways, T cell receptor signaling, Jak-STAT signaling were selected as group leading terms, as shown in Figure 5. Indeed, these group leading terms were either experimentally or computationally found to be related with RA, as can be seen in Table 2. This experiment generated the interconnections between the pathways that were found to be related with RA in our analysis.

**Figure 6:**
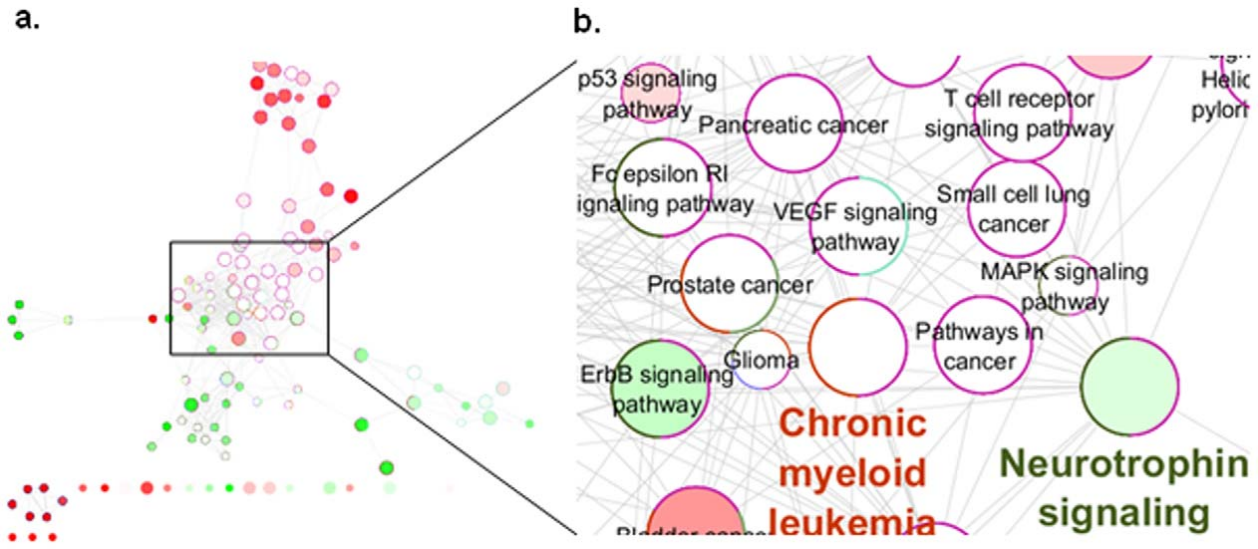
Comparison of KEGG pathway terms with literature verified RA genes/our gene set were shown in green/red, respectively. Nodes represent the identified pathway terms from any one of the two sets. The color gradient showed the gene proportion of each set associated with the term. White color represented equal proportions from the two comparison sets. The size of the nodes reflected the statistical significance of the terms (term p-values corrected with Bonferroni). Following the convention in Figure 4, edges represented the existence of the shared genes between the pathway terms and node border colors mapped to the group colors. Zoomed in view of panel a is shown in panel b.

To further check for the biological significance of our results, we compared the functional enrichments of the genes found in the highest scoring active sub-network with the functional enrichments of previously determined 331 genes verified by either OMIM database or by literature retrieved from the NCBI PubMed module to be associated with RA [61]. While our highest scoring sub-network with 275 genes enriched for 87 KEGG pathways, these 331 genes mapped to 88 pathways. Among those, 37 pathways were found in common, showing significant overlap between pathways coming from our study and the literature. In Figure 6a, the different proportion of the genes found in KEGG pathways from two sets was represented with a color gradient from green for literature verified RA genes, to red for our gene set. White denoted the pathways found in both sets with equal number of genes. As shown in Figure 6b (the zoomed in view), Pathways in cancer, T cell receptor signaling pathway, MAPK signaling pathway were found in both sets with the contribution of equal number of genes (shown in white). Whereas, the light green color in Neurotrophin signaling pathway term indicated that although most of the RA associated genes in this pathway comes from literature verified set, some of the genes in our gene set were assigned to this pathway.

## Discussion

Many reports of the genome wide associaton studies emerging in the literature, and the online GWAS catalog (http://www.genome.gov/26525384), including 273 published GWAS so far by National Human Genome Research Institute (NHGRI), are the clear evidences of the success of GWAS. Unfortunately, using the traditional approaches in GWAS, only the strongest associations can be detected; and there are many more SNPs/genes still to be found as associated with disease [66], [67]. Lately, several GWAS [8], [9], [10], [11], [12] have proposed the use of prior knowledge in the form of pathway databases, such as the KEGG and Biocarta, or gene ontology databases. On the other hand, Franke et al. [13] suggested the use of protein interaction network information along with pathway-based analysis. For Multiple Sclerosis GWAS data, Baranzini et al. [5] demonstrated the utility of network-based analysis. On top of these pathway and network based analyses of GWAS, here we devised a methodology that also integrates the functional information of a SNP as a third component. As a result of this multidimensional screening approach, our methodology generated a comprehensive list of functionally important KEGG pathways for RA (Table 2). While most of these associations can be thought as computational predictions, the functional relations of five of these pathways (Jak-STAT signalling, apoptosis, T cell receptor signalling, leukocyte transendothelial migration and cytokine-cytokine receptor interaction) with RA pathogenesis are shown in the reviews by Raychaudhuri and Plenge et al. [41], [42], [46].

Additionally, the effect of Toll-like receptor (TLR) signaling pathway and MAPK signaling pathway on RA is known as following: TLRs are membrane-bound receptors which are expressed in innate immune cells, such as macrophages and dendritic cells. TLRs signaling plays an important role in the activation and direction of the adaptive immune system by the up-regulation of co-stimulatory molecules of antigen presenting cells. The activation of the TLRs signaling pathway can trigger the activation of the MAPK and NF-kB pathways. Evidence is emerging that certain TLRs play a role in the pathogenesis of infectious and/or infammatory diseases. There is considerable evidence from rodent models that activation of the TLRs can induce or exacerbate inflammatory arthritis [68].

The role of MAPK signaling pathway in the development and progress of RA was shown to be related to cartilage damage, which is a hallmark of RA. Cartilage damage is based on increased proteoglycan loss as well as attachment and invasion of inflammatory tissue into the cartilage, which leads to its structural disintegration. Production of matrix metalloproteinases (MMPs) by synovial tissue appears to be a key prerequisite for synovial tissue to invade and destroy cartilage. MAPK is a crucial signal transduction pathway for inflammation and carries information about inflammatory stimuli to the cell nucleus. Synthesis of MMPs is regulated through multiple MAPK families, suggesting that a blockade of MAPK might have structural benefit in arthritis [43], [69]. Also, activation of stress kinase pathways ERK, JNK, and p38 MAPK is a typical feature of chronic synovitis during RA, and several proinflammatory mediators use the signaling of these stress kinase pathways [70].

Cytokine-cytokine receptor interaction pathway has been previously identified by two other studies as RA associated and included in the KEGG functional enrichment of known disease genes [64], [65]. Even though this term has not been found as significant in our highest scoring sub-network, it has been identified in the functional enrichment of our third highest scoring sub-network. Due to the nature of the search algorithm used by jActive Modules, several of the identified sub-networks overlap extensively in their component genes. Since it is complicated and cumbersome to represent the enrichment analysis of all identified sub-networks, here we have shown only the results from our highest scoring sub-network. In future, we aim to visualize the KEGG enrichment analysis results from all identified 5 top scoring sub-networks in a comprehensive manner.

To test whether the identified KEGG pathways could be obtained by chance, we tested the enrichment in KEGG pathways for 100 randomly generated networks of size 275. The enrichment of these 100 random networks returned 68 different KEGG pathways. Among those 68 pathways, only two of the KEGG pathways (Type I diabetes mellitus and Allograft rejection) overlap with the pathways shown in the Tables 2 and 3. However, the statistical significance of these pathways were low (term p-values = 0,013 and 0,007 respectively). These two pathways are found only for one random network out of 100 randomly generated networks and both pathways are found due to the existence of the following 5 random genes in this network, i.e. PRF1, HLA-B, FAS, HLA-DQA1, IL2. Whereas in our pathway analysis (as shown in Table 2), more genes are identified as part of Type I diabetes mellitus and Allograft rejection pathways (i.e. CD28, HLA-B, HLA-C, HLA-DMB, HLA-DPA1, HLA-DQA2, HLA-DRA, IL12A). Hence, our gene list includes different genes compared to the ones found in random network with higher significance (term p-values = 6,24E-03 and 2,16E-03 respectively). The detailed result of this experiment can be found in Supplementary Table S4.

Since only a couple of KEGG pathways are known to be associated with RA in literature, for verification purposes we also compared the genes as part of these pathways with the drug target genes of RA in Pharmaccogenomics Knowledge Base. To this end, we tried to find out whether taking the genes in pathway context would enhance the results of GWA study by identifying additional target genes. As result of assigning SNPs coming from GWAS to genes we identified 4094 genes. Only 14 of them were mapped to 83 RA specific drug target genes. Following the application of our method, we identified KEGG pathways that are affected by the SNPs, and these pathways contained 25 out of 83 RA specific drug target genes (listed in Supplementary Table S3). This provided an added value to GWAS analysis showing that not only the genes affected by the SNPs may be the drug targets but also other genes in these affected pathways may also be the drug targets, as shown by 11 extra genes identified. The analysis of SNP affected genes in a pathway context provides added value in identification of potential drug targets.

It is noteworthy to mention that pathway-based analyses, like it is presented here, are limited to our knowledge of cellular processes. The biological functions of most of the genes in the genome are not known. Since network and pathway tools make use of functional information from gene and protein databases, they are biased toward the well-studied genes, interactions, and pathways. Also, variants associated to genes not represented in the protein-protein interaction network were not evaluated in this analysis. Nevertheless, there is scope for the development of related methodologies to increase the power to detect associations in these genes. By combining information from several sources (functional properties of SNPs, genetic association of a SNP with the disease, PPI network), as shown in this paper, such limitations can be overcome. We also would like to point out that our method is not intended to be used for tag SNPs which are associated with a specific phenotype. As a future work, we plan to fully automate our method and convert to a webserver such that takes GWAS data as an input and generates disease specific pathway terms.

In summary, in this article we described a network and pathway-oriented analysis of GWAS data that also incorporates functional features of a SNP. In order to determine the biological significance of our results, we compared our findings with RA associated gene list obtained from OMIM database, or retrieved from literature using the NCBI PubMed module, or downloaded from Pharmaccogenomics Knowledge Base website. The main contributions of this paper can be summarized as follows:

1. We present a novel pathway and network oriented GWAS analysis that challenges to identify disease associated KEGG pathways by combining nominally significant evidence of genetic association with current knowledge of biochemical pathways, protein-protein interaction networks, and functional information of selected SNPs.
2. We identified additional KEGG pathways (e.g. Pathways in cancer, Neurotrophin signaling, Chemokine signaling pathways) as associated with RA. Furthermore, the KEGG functional enrichment of drug target genes included these terms.
3. Among the previously identified pathways, we identified additional genes associated with RA (e.g. Antigen processing and presentation, Tight junction). Importantly, within these pathways, the associations between some of these additionally found genes, such as HLA-C, HLA-G, PRKCQ, PRKCZ, TAP1, TAP2 and RA were verified by either OMIM database or by literature retrieved from the NCBI PubMed module.
4. Since our method can be easily applied to GWAS datasets of other diseases, it will facilitate the identification of disease specific pathways; and hence accelerate the development of more specific and useful drugs with less side effects.

To conclude, our results show that incorporating SNP functional properties, protein-protein interaction networks, pathway classification tools into GWAS can dissect leading molecular pathways, which cannot be picked up using traditional analyses. For GWAS analysis of complex diseases, novel disease-susceptibility genes and mechanisms can only be identified by looking beyond the tip of the iceberg (the most significant SNPs/genes). The development of pathway and network-based approaches that also integrate prior biological knowledge for mining the associations of a group of SNPs, will take us one step closer to unravel the complex genetic structure of common diseases.

## Supporting Information

Table S1 Complete list of drug target genes for RA, downloaded from Pharmaccogenomics Knowledge Base website is shown in Table S1.
(TXT)

Table S2 Complete list of the KEGG functional enrichment of drug target genes for RA is shown in Table S2.
(TXT)

Table S3 List of 25 drug target genes that are found to be associated with RA when all the genes in the identified KEGG pathways are considered.
(TXT)

Table S4 KEGG functional enrichment results for 100 randomly generated sub-networks is shown in Table S4.
(XLSX)
